# Errata

**Published:** 2010-07

In the January issue of this journal [*J Ophthalmic Vis Res* 2010; 5(1)], two errors were found regarding author names:

In the Photo Essay, “Bilateral Orbital Cavernous Hemangiomas” on page 65 ( *J Ophthalmic Vis Res* 2010; 5(1): 65–67), the author Reza Erfanian-Salim was misspelled as Mohammad-Reza Erfanian. The correct authors should read:

**Maryam Aletaha, MD; Reza Erfanian-Salim, MD; Abbas Bagheri, MD**

**Hossein Salour, MD; Mohammad Abrishami, MD**

In the Original Article, “Higher-Order Aberrations in Myopic Eyes” on page 3 ( *J Ophthalmic Vis Res* 2010; 5(1): 3–9), the author Azade Doozande was omitted. The correct authors should read:

**Farid Karimian, MD; Sepehr Feizi, MD; Azade Doozande, MD**

